# Squamous cell carcinoma of the uterine cervix associated with osteoclast-like giant cells: A case report and review of the literature

**DOI:** 10.3892/ol.2014.2384

**Published:** 2014-07-25

**Authors:** GUOHUA YU, CHUNHUA LIN, WEI WANG, YEKUN HAN, GUIMEI QU, TINGGUO ZHANG

**Affiliations:** 1 Department of Pathology, Shandong University School of Medicine, Jinan, Shandong 250012, P.R. China; 2 Department of Pathology, Affiliated Yantai Yuhuangding Hospital, Medical College of Qingdao University, Yantai, Shangdong 264000, P.R. China; 3 Department of Surgery, Affiliated Yantai Yuhuangding Hospital, Medical College of Qingdao University, Yantai, Shangdong 264000, P.R. China; 4 Department of Pharmacology, Affiliated Hospital, Binzhou Medical College, Binzhou, Shandong 256603, P.R. China

**Keywords:** uterine cervix, squamous cell carcinoma, osteoclast-like giant cells

## Abstract

Squamous cell carcinoma is a common malignant tumor of the uterine cervix. The present study reports the case of squamous cell carcinoma of the uterine cervix with osteoclast-like giant cells (OGCs) in an 84-year-old female who had suffered from irregular vaginal bleeding for one month. Colposcopy was performed and a cauliflower-like mass was identified in the front lip of the uterine cervix. Biopsy was then performed, and the tumor was found to be composed of epithelial cell nests, ranging in size. The neoplastic cells exhibited unclear boundaries and eosinophilic cytoplasm. Additionally, the nuclei were atypical and mitosis was observed. Among the epithelial nests, there were numerous OGCs with abundant eosinophilic cytoplasm, as well as multinucleation with bland nuclei. By immunohistochemical staining, the epithelial cells were positive for cytokeratin, while negative for CD68 and vimentin. By contrast, the immunophenotype of the OGCs was the exact opposite. Based on the histological characters, a diagnosis of squamous cell carcinoma of the uterine cervix associated with OGCs was made. Considering the age of the patient, radiotherapy was administered. The patient succumbed to brain metastasis of the tumor after eight months of follow-up.

## Introduction

Osteoclast-like giant cells (OGCs) have been described in numerous types of malignant tumors, such as pancreatic, breast, renal, skin, stomach and urinary bladder (1–6). However, OGCs have rarely been found in association with uterine cervix neoplasm. To the best of our knowledge, only three cases of squamous cell carcinoma of the uterine cervix with OGCs have been reported in the English literature (7,8). In the three previous cases, all patients were elderly females (>60 years old) and irregular postmenopausal bleeding was the most common clinical symptom observed. The three patients succumbed to the disease within 14 months of surgery or treatment with radiochemotherapy (7,8). Therefore, the origination and prognostic significance of OGCs in squamous cell carcinoma of uterine cervix are poorly understood. The present study reports a fourth case of squamous cell carcinoma of the uterine cervix associated with OGCs, and the clinicopathological features are discussed. In this study, the case of an 84-year-old female with irregular vaginal bleeding is presented. A cauliflower-like mass was identified in the front lip of the uterine cervix by colposcopy. The patient received palliative radiotherapy and succumbed to brain metastasis after eight months of follow-up. Written informed consent was obtained from the patient for publication of this case report and any accompanying images.

## Case report

### Clinical data

An 84-year-old post-menopausal female presented to the Department of Gynaecology at Laishan People’s Hospital (Yantai, China) with irregular vaginal bleeding of one month, and was then sent to the Department of Gynaecology at the Affiliated Yantai Yuhuangding Hospital (Yantai, China). Colposcopy was performed and a cauliflower-like mass was identified in the front lip of the uterine cervix. The neoplasm was ~5 cm in diameter and fragile in texture. Abdominal ultrasound examination showed multiple swollen lymph nodes surrounding the great vessels. The blood counts, clinical biochemistry, urine analysis and endocrine profile were all within normal ranges.

### Pathological findings

**Figure 1:**
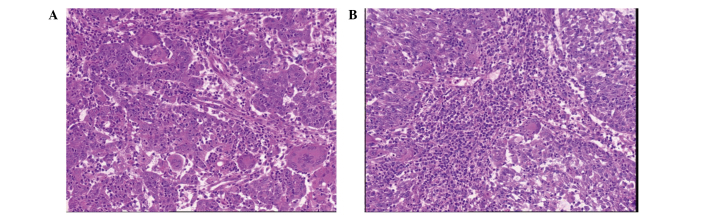
Histological features of the tumor. (A) Irregular epithelial cell nests show infiltrative growth, and there are numerous OGCs among the nests. The nuclei of the epithelial cells are atypical and mitosis is evident, while the nuclei of the OGCs are bland and monomorphic. (B) Numerous lymphatic cells are positive for CD3, mostly among the epithelial cell nests and neoplastic stroma, as shown by immunohistochemical staining. Hematoxylin and eosin staining; magnification, ×100. OGCs, osteoclast-like giant cells.

Biopsy was performed and histological findings showed that epithelial cell nests, ranging in size, had infiltrated the stroma. The neoplastic cells presented unclear boundaries and eosinophilic cytoplasm. In addition, the nuclei were atypical and mitosis was observed. There were no obvious keratin pearls or intercellular bridges. Among the epithelial cell nests, there were numerous OGCs with abundant eosinophilic cytoplasm and multinucleation. The nuclei were bland, monomorphic and round or oval in shape. The absence of features of cytological atypia and mitosis distinguished these nuclei from those of the neoplastic cells (Fig. 1A). There were numerous lymphatic cells in the epithelial cell nests and neoplastic stroma (Fig. 1B).

### Immunohistochemical findings

**Figure 2:**
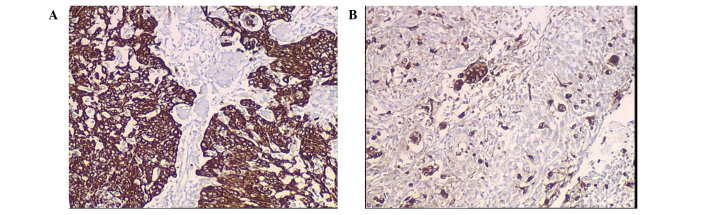
Immunohistochemical features of the OGCs. The OGCs are (A) negative for cytokeratin and (B) positive for CD68 (magnification, ×100). OGCs, osteoclast-like giant cells.

Using immunohistochemical techniques, the neoplastic cells were identified to be positive for cytokeratin (Fig. 2A); whereas the OGCs were positive for CD68 (Fig. 2B) and vimentin, but negative for cytokeratin. The majority of lymphatic cells were positive for CD3 and only a small number of lymphocytes expressed CD20. On the basis of the clinical manifestations, histological features and immunohistochemical findings, a diagnosis of non-keratinizing squamous carcinoma of the uterine cervix with OGCs was made. With consideration of the patient’s age, the surgical treatment was cancelled with the patient’s consent and radiotherapy was administered instead. The total follow-up period was eight months. The patient was alive and the size of neoplasm had decreased prior to the end of radiotherapy, however, the patient succumbed to brain metastasis of the tumor at eight months of follow-up.

## Discussion

A number of studies have shown that OGCs are a unique type of microphage emerging from the mesenchymal tissue, with similar histological features to osteoclasts (3,5,9,10). However, it was previously thought that OGCs were a type of tumor cell that originated from the epithelium (2). In the present case, the OGCs were bland and lacked atypia. Immunohistochemically, the OGCs were positive for CD68 and vimentin, but negative for cytokeratin. These results support the hypothesis that OGCs may simply be immunoreactive cells derived from the mesenchymal tissue. In this case, it is important to distinguish OGCs from multinucleated cells, which are known to indicate a poor prognosis (11). The cells of giant cell tumors exhibit nuclei with hyperchromatism and pleomorphism, while OGCs are an immunoreactive component with uniform chromatin and no obvious atypia (11).

OGCs have been described in numerous types of malignant tumors, such as pancreatic, breast, renal, skin, stomach and urinary bladder (1–6). The reason for the association of OGCs with these tumor types may be the chemotaxis of tumor cells or inflammatory cells. In the present case, numerous lymphocytes, among which T lymphocytes were predominant, were identified in the epithelial cell nests and the neoplastic stroma. Therefore, this finding adds support to the abovementioned theory.

To the best of our knowledge, only three cases of squamous cell carcinoma associated with OGCs in the uterine cervix have been reported (7,8), and the clinical information is presented in Table I. All the patients were elderly females >60 years of age, and irregular post-menopausal bleeding was the common clinical manifestation. In three cases, the mass showed extruding growth, while one case showed an infiltrated neoplasm. The tumor diameters ranged from 4.5 to 6 cm, and the clinical stage was Ib in all cases according to the International Federation of Gynecology and Obstetrics staging for carcinoma of the vulva, cervix, and endometrium (2009) (12). The type of squamous cell carcinoma which was accompanied by OGCs was sarcomatoid type or non-keratinizing type, which are poorly differentiated types of squamous cell carcinoma. Three patients died within 14 months of surgery or radio-chemotherapy. One patient survived, but was followed up for a shorter time period. Although OGCs may be immunoreactive cells, the presence of OGCs in squamous cell carcinoma of the uterine cervix appears to be an indicator of poor prognosis. However, the type of primary tumor appears to be the main determinant of prognosis, and additional clinical data are required to determine the significance of these findings.

## Figures and Tables

**Table I:** Previously reported cases of squamous cell carcinoma associated with OGCs in the uterine cervix.

Case	Author (ref.)	Age, years	Tumor diameter, cm	Tumor stage	Clinical manifestation	Growth pattern	Histological type of SCC	Immumohistochemical staining of OGC	Treatment	Follow-up/Status
1	Pang (7)	65	6	Ib	IVB	Polypoid mass	Sarcomatoid type	Positive for Kp1 and CD68; negative for CK and vimentin	Hysterectomy and lymphadenectomy; radiotherapy and chemotherapy	7 weeks, DOD
2	Pang (7)	61	5	Ib	IVB	Polypoid mass	Sarcomatoid type	Positive for Kp1 and CD68; negative for CK and vimentin	Hysterectomy and lymphadenectomy; radiotherapy and chemotherapy	14 months, DOD
3	Singh *et al* (8)	60	4.5	Ib	IVB	Infiltrated mass	NK type	Positive for CD68; negative for CK, EMA and vimentin	Radiotherapy and chemotherapy	6 months, ACR
4	Present case	84	5	Ib	IVB	Polypoid mass	NK type	Positive for CD68 and vimentin; negative for CK	Radiotherapy	8 months, DOD

Tumor stage was diagnosed in accordance with the International Federation of Gynecology and Obstetrics staging for staging for carcinoma of the vulva, cervix, and endometrium (2009) (12). OGCs, osteoclast-like giant cells; IVB, irregular vaginal bleeding; SCC, squamous cell carcinoma; NK, non-keratinizing; DOD, dead of disease; ACR, alive with complete regression of the growth.
